# Positive allosteric adenosine A_2A_ receptor modulation suppresses insomnia associated with mania- and schizophrenia-like behaviors in mice

**DOI:** 10.3389/fphar.2023.1138666

**Published:** 2023-04-19

**Authors:** Yang Lin, Koustav Roy, Shuji Ioka, Rintaro Otani, Mao Amezawa, Yukiko Ishikawa, Yoan Cherasse, Mahesh K. Kaushik, Daniela Klewe-Nebenius, Li Zhou, Masashi Yanagisawa, Yo Oishi, Tsuyoshi Saitoh, Michael Lazarus

**Affiliations:** ^1^ International Institute for Integrative Sleep Medicine (WPI-IIIS), Tsukuba, Ibaraki, Japan; ^2^ Institute of Medicine, University of Tsukuba, Tsukuba, Ibaraki, Japan

**Keywords:** sleep disorder, REM sleep, wakefulness, dopamine, neuroleptics, EEG, Map6 (mouse), stable tubule-only polypeptide

## Abstract

**Background:** Insomnia is associated with psychiatric illnesses such as bipolar disorder or schizophrenia. Treating insomnia improves psychotic symptoms severity, quality of life, and functional outcomes. Patients with psychiatric disorders are often dissatisfied with the available therapeutic options for their insomnia. In contrast, positive allosteric modulation of adenosine A_2A_ receptors (A_2A_Rs) leads to slow-wave sleep without cardiovascular side effects in contrast to A_2A_R agonists.

**Methods:** We investigated the hypnotic effects of A_2A_R positive allosteric modulators (PAMs) in mice with mania-like behavior produced by ablating GABAergic neurons in the ventral medial midbrain/pons area and in a mouse model of schizophrenia by knocking out of microtubule-associated protein 6. We also compared the properties of sleep induced by A_2A_R PAMs in mice with mania-like behavior with those induced by DORA-22, a dual orexin receptor antagonist that improves sleep in pre-clinical models, and the benzodiazepine diazepam.

**Results:** A_2A_R PAMs suppress insomnia associated with mania- or schizophrenia-like behaviors in mice. A_2A_R PAM-mediated suppression of insomnia in mice with mania-like behavior was similar to that mediated by DORA-22, and, unlike diazepam, did not result in abnormal sleep.

**Conclusion:** A_2A_R allosteric modulation may represent a new therapeutic avenue for sleep disruption associated with bipolar disorder or psychosis.

## 1 Introduction

Insomnia is often associated with severe psychiatric illnesses such as bipolar disorder or schizophrenia (Soehner et al., 2013). Bipolar disorder, formerly called manic depression, affects approximately 1% of people worldwide, and is characterized by periods of depression and periods of abnormally elevated mood (mania) lasting days to weeks. Currently available treatments are not targeted and consist mainly of mood stabilizers such as lithium in combination with antidepressants or antipsychotics. These drugs, however, are associated with a variety of side effects, such as trouble walking, muscle weakness, psychosis, extrapyramidal side effects, and sexual dysfunction (Zarate, 2000; Gitlin, 2016). The neuropsychiatric disorder schizophrenia is characterized by positive symptoms (hallucinations, delusions, disorganized speech and behavior, and agitated body movements), negative symptoms (deficits in affective and social domains), and cognitive impairment (disrupted attention, working memory, and executive function) (Ross et al., 2006). Schizophrenia also affects approximately 1% of individuals worldwide. The age of onset is typically between 15 and 25 years of age (Schultz et al., 2007). A growing body of evidence indicates that sleep disorders negatively affect the course of psychiatric illness and contribute to functional impairments. When psychiatric disorders are successfully treated, associated sleep disorders such as insomnia often do not improve. On the other hand, treating insomnia improves psychotic symptoms severity, quality of life, and functional outcomes (Kaskie et al., 2017).

Benzodiazepines and Z-drugs, which induce sleep by enhancing signaling of the inhibitory neurotransmitter γ-aminobutyric acid (GABA), are the most commonly prescribed first-line agents for the treatment of insomnia (Wafford and Ebert, 2008). These medications, however, are plagued by a wide range of disadvantages and safety issues: benzodiazepine-like drugs worsen sleep quality by increasing light sleep at the expense of healthy deep sleep and rapid eye movement sleep (REMS). In addition, the use of benzodiazepine drugs is limited by their side effects, such as next-day sleepiness, cognitive impairment, amnesic effects, changes in appetite, and their long-term administration can lead to the development of tolerance and dependence (Vgontzas et al., 1995; Aragona, 2000). Z-Drug ingestion can also lead to a psychedelic state that increases the risk of car accidents and suicide (Hemmelgarn et al., 1997; Ghosh et al., 2020). In addition, orexin receptor antagonists were developed and approved for the treatment of insomnia (Cox et al., 2010). Major issues with these drugs, however, include next-morning sleepiness with possible signs of muscle weakness, weird dreams, sleepwalking, other nighttime behaviors, or suicidal ideation (Jacobson et al., 2014). Moreover, a recent rodent study suggested that chronic use of orexin antagonists may induce narcolepsy-like symptoms (Kaushik et al., 2021). Patients with psychiatric disorders are often dissatisfied with most available therapeutic options.

The neuromodulator adenosine enhances drowsiness by binding to A_2A_ receptors (A_2A_Rs). Because A_2A_Rs are widely expressed in the cardiovascular system, direct use of A_2A_R agonists can have adverse cardiovascular effects and is thus not considered for therapeutic application. An A_2A_R positive allosteric modulator (PAM), 3,4-difluoro-2-((2-fluoro-4-iodophenyl)amino)benzoic acid (A_2A_RPAM-1), was recently identified to allosterically modulate A_2A_Rs, thereby enhancing extracellular adenosine signaling and inducing slow-wave sleep (SWS) without cardiovascular side effects in wild-type (WT) mice (Korkutata et al., 2019). Whether the hypnotic effects of A_2A_RPAM-1 are also observed in mouse models of psychiatric illness, however, is unclear. In the present study, we used a mouse exhibiting mania-like behaviors in which the GABAergic neurons of the ventral medial midbrain/pons (VMP) area were selectively ablated by stereotaxic microinjection of adeno-associated virus (AAV) expressing Cre-dependent diphtheria toxin subunit A (DTA) into vesicular GABA transporter (VGAT, a marker of GABAergic neurons)-Cre mice (Takata et al., 2018; Honda et al., 2020). We evaluated the hypnotic effects of the A_2A_R PAM A_2A_RPAM-1 and a structurally related compound, A_2A_RPAM-2, in mice with mania-like behavior and compared the sleep properties of the A_2A_R PAMs with DORA-22, a dual orexin receptor antagonist that improves sleep in pre-clinical models, and the benzodiazepine diazepam. In addition, as the microtubule-associated protein 6 (MAP6, also known as stable-only polypeptide, STOP) gene is associated with schizophrenia (Shimizu et al., 2006) and the MAP6 knockout (KO) mouse is a genetic mouse model of schizophrenia/psychosis (Andrieux et al., 2002; Profitt et al., 2016), we further investigated the hypnotic effects of A_2A_RPAM-1 in MAP6 (STOP) KO mice.

## 2 Results

### 2.1 Intraperitoneal administration of A_2A_R PAMs induced physiologic sleep in mice with mania-like behavior

**FIGURE 1:**
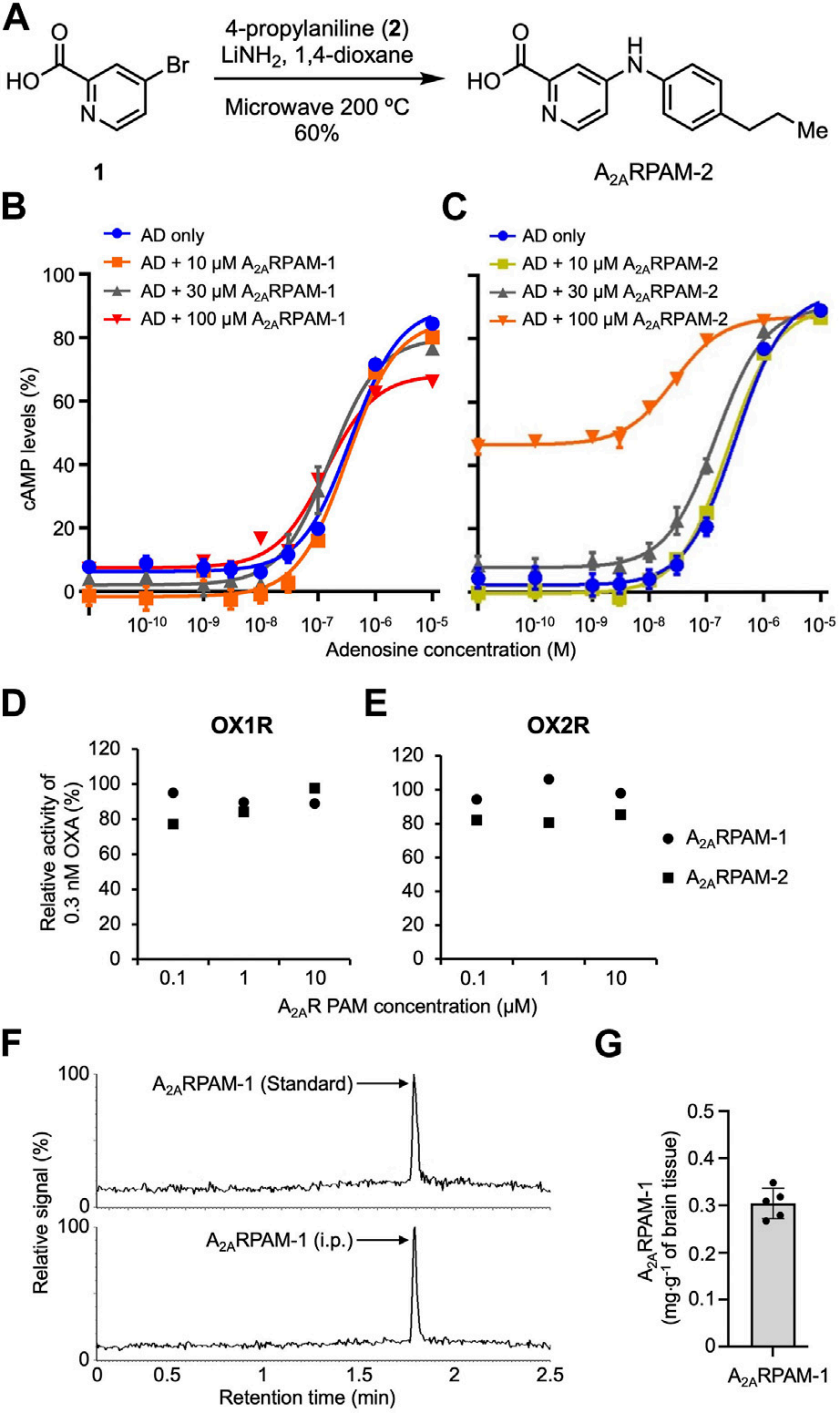
The monocarboxylic acids A_2A_RPAM-1 and A_2A_RPAM-2 are positive adenosine A_2A_ receptor allosteric modulators. **(A)** Chemical synthesis of A_2A_RPAM-2. A_2A_RPAM-2 was produced by combining 4-bromo-2-pyridinecarboxylic acid (1) and 4-propylaniline (2). **(B,C)** Dose-dependent changes of cAMP levels in mA_2A_R-expressing Chinese hamster ovary cells after treatment with various adenosine (AD) and A_2A_RPAM-1 **(B)** or A_2A_RPAM-2 **(C)** concentrations. Experiments were performed in triplicate wells for each condition and data are presented as mean ± SEM. **(D,E)** Antagonistic activity of A_2A_RPAM-1 and A_2A_RPAM-2 against human orexin A (OXA) at orexin-1 receptors (D, OX_1_R) and orexin-2 receptors (E, OX_2_R) in a cell-based calcium assay. Experiments were performed in duplicate wells for each condition and data are presented as mean ± SEM. **(F,G)** UPLC-MS/MS analysis of brain samples from mice injected intraperitoneally with 75 mg kg^-1^ A_2A_RPAM-1. Single-ion (m/z 392) signals **(F)** and total brain concentrations **(G)** of A_2A_RPAM-1 in the brain samples. Data (n = 5) are presented as mean ± SEM.

To test the effects of intraperitoneal (i.p.) injection of A_2A_RPAMs on sleep/wake behavior in mice with mania-like behavior and control mice, we used A_2A_RPAM-1 and the newly developed A_2_ARPAM-2, which is a monocarboxylic acid structurally related to A_2A_RPAM-1 that was identified in a structure-activity relationship screen for A_2A_RPAM-1 to have stronger allosteric activity in Chinese hamster ovary (CHO) cells expressing mouse A_2A_Rs (Figures 1B, C). Orexin receptor antagonists are used for the treatment of insomnia (Cox et al., 2010); therefore, the antagonistic activity of A_2A_RPAM-1 and A_2A_RPAM-2 against human orexin A (OXA) at orexin-1 receptors (OX_1_Rs) and orexin-2 receptors (OX_2_Rs) was measured in a cell-based calcium assay. A_2A_RPAM-1 and A_2A_RPAM-2 did not show physiologically relevant inhibition of OXA activity at OX_1_Rs (Figure 1D) and OX_2_Rs (Figure 1E). We also analyzed whether the A_2A_RPAM-1 crosses the blood-brain barrier using ultra-high performance liquid chromatography coupled to tandem mass spectrometry (UPLC-MS/MS). One hour after administering 75 mg kg^−1^ A_2A_RPAM-1 (i.p.), we detected the compound in the brain at an average level of 0.3 mg g^-1^ of brain tissue (Figures 1F,G).

**FIGURE 2:**
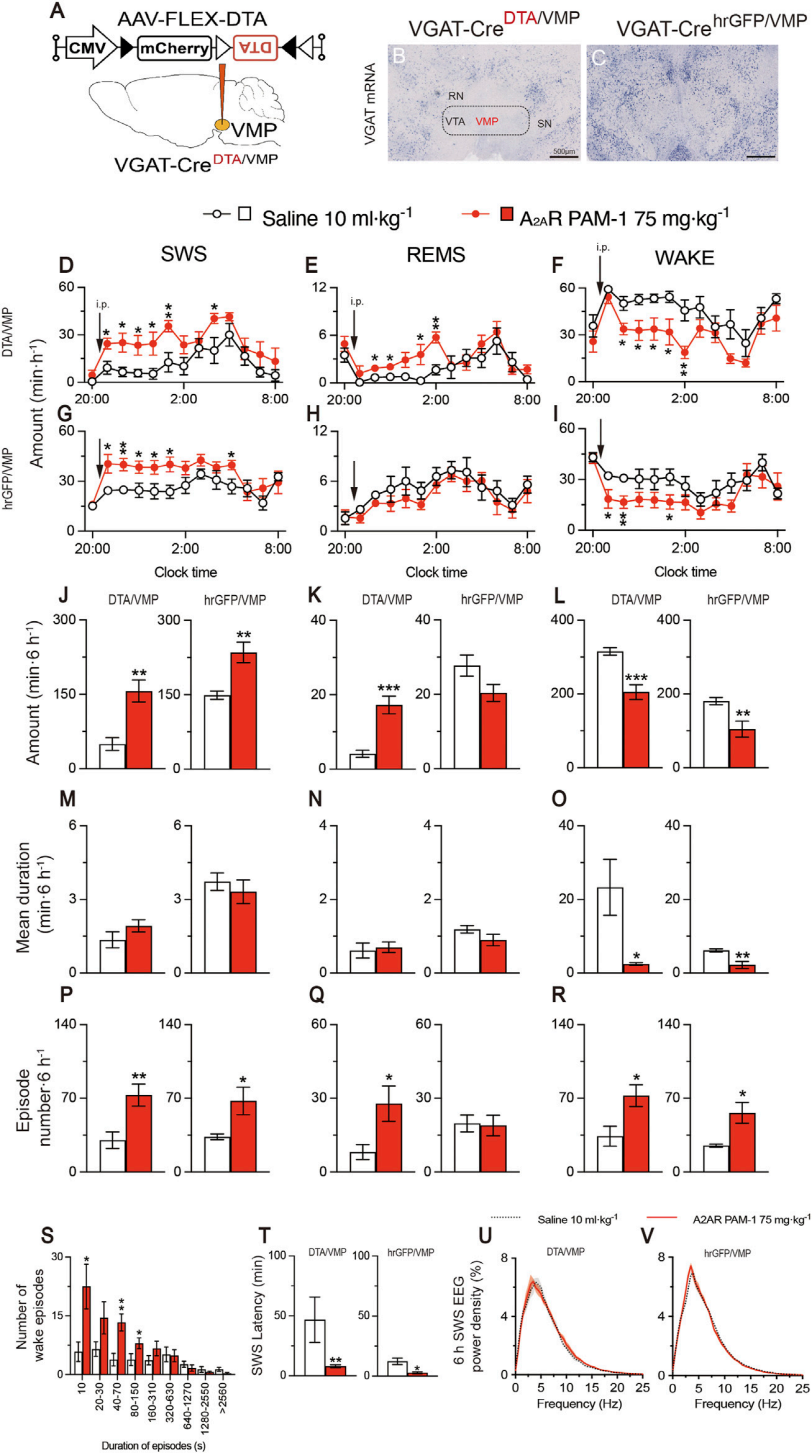
Intraperitoneal administration of A_2A_R PAM-1 induces sleep in mice with mania-like behavior. **(A)** VGAT-Cre mice were injected with AAV-FLEX-DTA (VGAT-Cre^DTA/VMP^ mice) or AAV-FLEX-hrGFP (VGAT-Cre^hrGFP/VMP^ mice, control) into the VMP region. **(B,C)** Brain sections stained against VGAT mRNA in VGAT-Cre^DTA/VMP^ **(B)** and VGAT-Cre^hrGFP/VMP^ **(C)** mice confirm that the VMP GABAergic neurons were ablated in VGAT-Cre mice. RN, red nucleus; SN, substantia nigra; VTA, ventral tegmental area. Scale bar, 500 μm. (D–R) Time course **(D–I)**, total amount **(J–L)**, mean duration **(M–O)**, and episode number **(P–R)** of SWS, REMS, and wakefulness in VGAT-Cre^DTA/VMP^ and VGAT-Cre^hrGFP/VMP^ mice after administration of saline or A_2A_RPAM-1. **(S)** Wake episode spectrum in VGAT-Cre^DTA/VMP^ mice. **(T)** Latency to sleep onset in VGAT-Cre^DTA/VMP^ and VGAT-Cre^hrGFP/VMP^ mice after administering saline or A_2A_RPAM-1. (U, V) SWS EEG power density for 6 h after injecting saline or A_2A_R PAM-1 into VGAT-Cre^DTA/VMP^ **(U)** and VGAT-Cre^hrGFP/VMP^ **(V)** mice. Data (n = 6/group) are presented as mean ± SEM. Significance levels are indicated as *p < 0.05, **p < 0.01, and ***p < 0.001.

**FIGURE 3:**
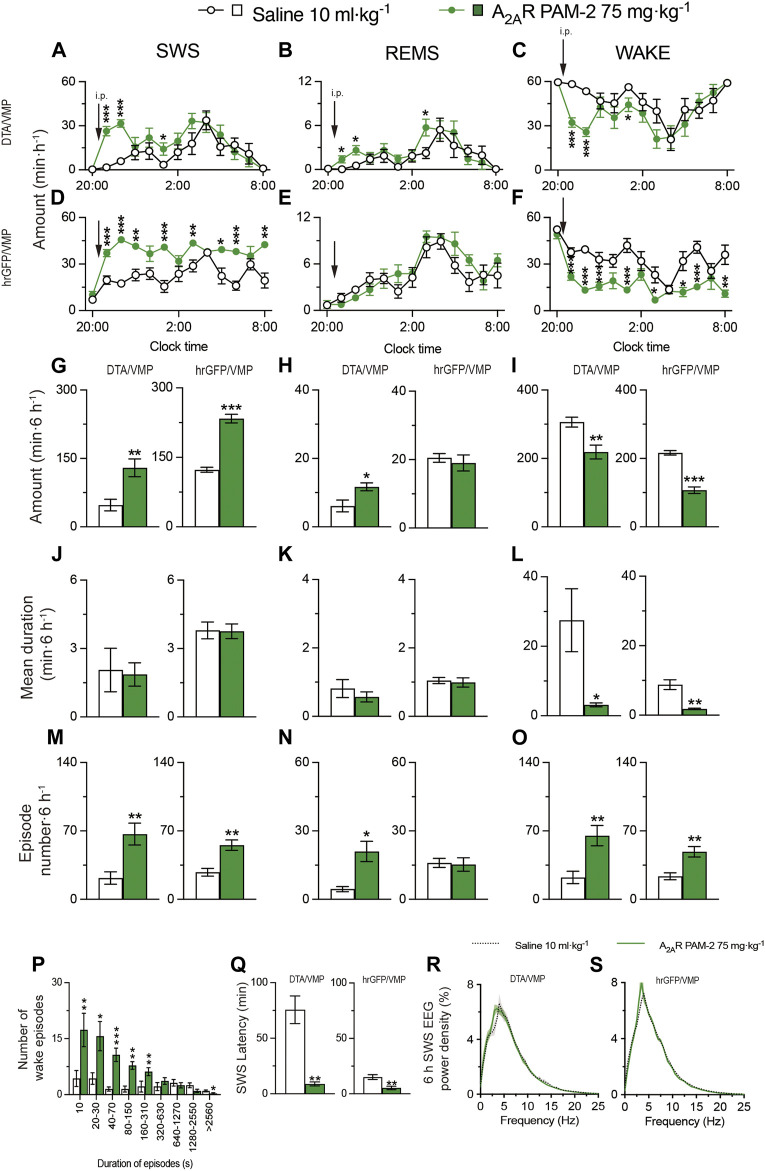
Intraperitoneal injection of A_2A_RPAM-2 induces sleep in mice with mania-like behavior. **(A–O)** Time course **(A–F)**, total amount **(G–I)**, mean duration **(J–L)**, and episode number **(M–O)** of SWS, REMS, and wakefulness in VGAT-Cre^DTA/VMP^ and VGAT-Cre^hrGFP/VMP^ mice after administering saline or A_2A_RPAM-2. **(P)** Wake episode spectrum in VGAT-Cre^DTA/VMP^ mice. **(Q)** Latency to sleep onset in VGAT-Cre^DTA/VMP^ and VGAT-Cre^hrGFP/VMP^ mice after administering saline or A_2A_RPAM-2. **(R,S)** SWS EEG power density for 6 h after injection of saline or A_2A_RPAM-2 into VGAT-Cre^DTA/VMP^ **(R)** and VGAT-Cre^hrGFP/VMP^ **(S)** mice. Data (n = 6/group) are presented as mean ± SEM. Significance levels are indicated as *p < 0.05, **p < 0.01, and ***p < 0.001.

To obtain mice with mania-like behavior, we selectively ablated VMP GABAergic neurons of VGAT-Cre mice with Cre-dependent viral expression of the DTA in the VMP (VGAT-Cre^DTA/VMP^ mice; Figures 2A, B) as previously reported (Takata et al., 2018; Honda et al., 2020). For controls, we prepared mice expressing humanized *Renilla reniformis*-derived green fluorescent protein (hrGFP) in VMP GABAergic neurons (VGAT-Cre^hrGFP/VMP^; Figure 2C). We analyzed electroencephalogram (EEG) and electromyogram (EMG) recordings made after injecting saline, A_2A_RPAM-1 (Figure 2), or A_2A_RPAM-2 (Figure 3) during the dark period at 21:00 when mice usually spend most of their time awake. Baseline sleep of the VGAT-Cre^DTA/VMP^ mice was significantly lower than that of VGAT-Cre^hrGFP/VMP^ mice, but SWS was greatly increased for 6 h after injecting A_2A_RPAM-1 (75 mg kg^−1^, i. p.) or A_2A_RPAM-2 (75 mg kg^−1^, i. p.) in VGAT-Cre^DTA/VMP^ mice, reaching the level of control mice (Figure 2D, SWS: *F*_(1, 10)_ = 15.45, *p* = 0.0028, ANOVA; Figure 2J left, *p* = 0.0022, Mann-Whitney U test; Figure 3A, *F*_(1, 10)_ = 5.808, *p* = 0.0367, ANOVA; Figure 3G left, *t*_(10)_ = 3.54, *p* = 0.0053; unpaired *t*-test). SWS was also increased in VGAT- Cre^hrGFP/VMP^ mice compared with saline-injected mice (Figure 2G, SWS: *F*_(1, 10)_ = 12.61, *p* = 0.0053, ANOVA; Figure 2J right, *t*_(10)_ = 3.86, *p* = 0.0031, unpaired *t*-test; Figure 3D, *F*_(1, 10)_ = 117.3, *p* < 0.0001, ANOVA; Figure 3G right, *t*_(10)_ = 10.3, *p* < 0.0001, unpaired *t*-test), similar to previously reported findings in WT mice (Korkutata et al., 2019). Interestingly, REMS was significantly induced by A_2A_RPAM-1 and A_2A_RPAM-2 in VGAT-Cre^DTA/VMP^ mice (Figure 2E, REMS: *F*_(1, 10)_ = 6.285, *p* = 0.0311, ANOVA; Figure 2K left, *t*_(10)_ = 5.09, *p* = 0.0005, unpaired *t*-test; Figure 3B, *F*_(1, 10)_ = 3.078, *p* = 0.1099, ANOVA; Figure 3H left, *t*_(10)_ = 2.69, *p* = 0.0225, unpaired *t*-test), but not in VGAT-Cre^hrGFP/VMP^ mice, the latter observation is similar to previous reports of WT mice (Korkutata et al., 2019).

Administration of A_2A_RPAM-1 (75 mg kg^−1^, i. p.) or A_2A_RPAM-2 (75 mg kg^−1^, i. p.) to VGAT-Cre^DTA/VMP^ and VGAT-Cre^hrGFP/VMP^ mice significantly increased the SWS episode number for 6 h in the dark period (Figure 2P left, *t*_(10)_ = 3.247, *p* = 0.0088, unpaired *t*-test; Figure 2P right, *t*_(10)_ = 2.54, *p* = 0.0480, unpaired *t*-test; Figure 3M left, *t*_(10)_ = 3.49, *p* = 0.0057, unpaired *t*-test; Figure 3M right, *t*_(10)_ = 4.18, *p* = 0.0019, unpaired *t*-test), whereas the A_2A_R PAMs did not affect the mean episode duration. In contrast, the A_2A_R PAMs only increased the REMS episode number in the VGAT-Cre^DTA/VMP^ mice (Figure 2Q left, *t*_(10)_ = 2.53, *p* = 0.0300, unpaired *t*-test; Figure 2Q right, *t*_(10)_ = 0.154, *p* = 0.8806, unpaired *t*-test; Figure 3N left, *t*_(10)_ = 3.59, *p* = 0.0128, unpaired *t*-test; Figure 3N right, *t*_(10)_ = 0.187, *p* = 0.8554, unpaired *t*-test). Moreover, the mean wake episode duration and number decreased (Figure 2O left, *t*_(10)_ = 2.73, *p* = 0.0409, unpaired *t*-test; Figure 2O right, *t*_(10)_ = 3.75, *p* = 0.0038, unpaired *t*-test; Figure 3L left, *t*_(10)_ = 2.68, *p* = 0.0434, unpaired *t*-test; Figure 3L right, *t*_(10)_ = 4.87, *p* = 0.0041, unpaired *t*-test) in VGAT-Cre^DTA/VMP^ mice and increased (Figure 2R left, *t*_(10)_ = 2.74, *p* = 0.0209, unpaired *t*-test; Figure 2R right, *t*_(10)_ = 3.12, *p* = 0.0249 unpaired t-tests; Figure 3O left, *t*_(10)_ = 3.51, *p* = 0.0057, unpaired *t*-test; Figure 3O right, *t*_(10)_ = 3.88, *p* = 0.0031 unpaired *t*-test) in VGAT-Cre^hrGFP/VMP^ mice after the A_2A_R PAM injections. While VGAT-Cre^DTA/VMP^ mice exhibit an unusually high number of wake episodes with a duration of 10 min or more, a strong shift to short wake episodes with a duration of less than 150 or 310 s was observed in VGAT-Cre^DTA/VMP^ mice treated with A_2A_RPAM-1 (Figure 2S) or A_2A_RPAM-2 (Figure 3P), respectively. The latency to sleep onset, defined as the time from the A_2A_R PAM injection to the appearance of the first SWS episode lasting longer than 20 s, was significantly decreased in the VGAT-Cre^DTA/VMP^ and VGAT-Cre^hrGFP/VMP^ mice treated with A_2A_RPAM-1 (Figure 2T left, *p* = 0.0022, Mann-Whitney U test; Figure 2T right, *t*_(10)_ = 3.42, *p* = 0.0145 unpaired t-tests) or A_2A_RPAM-2 (Figure 3Q left, *t*_(10)_ = 5.31, *p* = 0.0028, unpaired *t*-test; Figure 3Q right, *t*_(10)_ = 3.62, *p* = 0.0047 unpaired *t*-test) compared with vehicle treatment. To assess whether EEG activity was altered by A_2A_R PAMs administration, we compared the normalized EEG power spectrum of SWS in mice with mania-like behavior and control mice treated with saline or the A_2A_R PAMs (Figures 2U,V and 3R,S). EEG activity in the frequency range of 0.5–25 Hz during SWS was indistinguishable between A_2A_RPAM-1 or A_2A_RPAM-2–induced and natural (saline injection) SWS. These data suggest that A_2A_RPAM-1 and A_2A_RPAM-2 induced physiologic sleep rather than aberrant sleep in mice with mania-like behavior. Taken together, the results suggest that positive A_2A_R allosteric modulation restores SWS and even REMS in mice with mania-like behavior.

### 2.2 Oral administration of the dual orexin receptor antagonist DORA-22 induced physiologic sleep in mice with mania-like behavior

**FIGURE 4:**
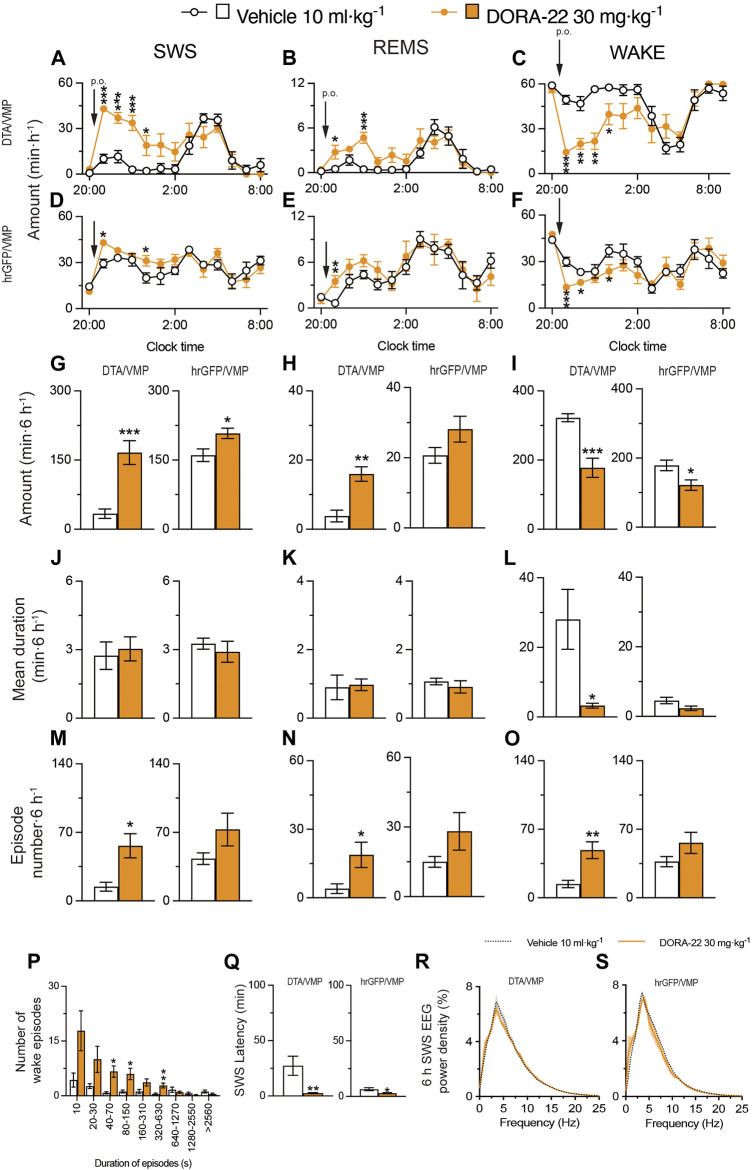
Oral administration of DORA-22 induces sleep in mice with mania-like behavior. **(A–O)** Time course **(A–F)**, total amount **(G–I)**, mean duration **(J–L)**, and episode number **(M–O)** of SWS, REMS, and wakefulness in VGAT-Cre^DTA/VMP^ and VGAT-Cre^hrGFP/VMP^ mice after administering vehicle or DORA-22. **(P)** Wake episode spectrum in VGAT-Cre^DTA/VMP^ mice. **(Q)** Latency to sleep onset in VGAT-Cre^DTA/VMP^ and VGAT-Cre^hrGFP/VMP^ mice after administering vehicle or DORA-22. **(R,S)** SWS EEG power density for 6 h after injection of vehicle or DORA-22 into VGAT-Cre^DTA/VMP^ **(R)** and VGAT-Cre^hrGFP/VMP^ **(S)** mice. Data (n = 6/group) are presented as mean ± SEM. Significance levels are indicated as *p < 0.05, **p < 0.01, and ***p < 0.001.

Next, based on a previously published study (Kaushik et al., 2021), we tested the effect of 30 mg kg^−1^ DORA-22 administered via oral gavage (p.o.) on sleep/wake behavior in mice with mania-like behavior and control mice (Figure 4). We analyzed EEG and EMG recordings made after the administration of vehicle or DORA-22 during the dark period at 21:00. SWS was increased for 6 h after DORA-22 administration in VGAT-Cre^DTA/VMP^ mice (Figure 4A, SWS: *F*_(1, 10)_ = 10.29, *p* = 0.0094, ANOVA; Figure 4G left, *t*_(10)_ = 4.76, *p* = 0.0008, unpaired *t*-test) and VGAT-Cre^hrGFP/VMP^ mice (Figure 4D SWS: *F*_(1, 130)_ = 4.215, *p* = 0.0421, ANOVA; Figure 4G right, *t*_(10)_ = 2.66, *p* = 0.0241, unpaired *t*-test) compared with vehicle treatment. REMS was significantly increased only in VGAT-Cre^DTA/VMP^ mice (Figure 4B, REMS: *F*_(1, 10)_ = 7.931, *p* = 0.0183, ANOVA; Figure 4H left, *t*_(10)_ = 4.50, *p* = 0.0011, unpaired *t*-test), whereas REMS tended to increase in the VGAT-Cre^hrGFP/VMP^ mice but the increase was not statistically significant. These results suggest that DORA-22 administration can restore a normal level of sleep in mice with mania-like behavior, similar to the A_2A_R PAMs. Oral administration of DORA-22 significantly increased the SWS and REMS episode numbers (Figure 4M left, *t*_(10)_ = 3.19, *p* = 0.0176, unpaired *t*-test; Figure 4N left, *p* = 0.0130, Mann-Whitney U test) but the episode duration was not increased significantly in mice with mania-like behavior (VGAT-Cre^DTA/VMP^ mice. The mean duration and number of SWS and REMS episodes were not significantly changed in the control mice (VGAT-Cre^hrGFP/VMP^ mice). The mean wake episode duration decreased (Figure 4L left, *t*_(10)_ = 2.87, *p* = 0.0345, unpaired *t*-test) and the mean wake episode number increased (Figure 4O left, *t*_(10)_ = 3.66, *p* = 0.0044, unpaired *t*-test) in VGAT-Cre^DTA/VMP^ mice after oral DORA-22 administration. A shift to shorter wake episodes was observed in VGAT-Cre^DTA/VMP^ mice treated with DORA-22 (Figure 4P). The latency to sleep onset was significantly decreased in the VGAT-Cre^DTA/VMP^ and VGAT-Cre^hrGFP/VMP^ mice treated with DORA-22 (Figure 4Q left, *p* = 0.0022, Mann-Whitney U test; Figure 4Q right, *t*_(10)_ = 2.41, *p* = 0.0370 unpaired t-tests) compared with vehicle treatment. To assess whether EEG activity was altered by oral DORA-22 administration, we compared the normalized EEG power spectrum of SWS in mice with mania-like behavior and control mice treated with vehicle or DORA-22 (Figures 4R,S). EEG activity in the frequency range of 0.5–25 Hz during SWS was indistinguishable between DORA-22–induced and natural (vehicle injection) SWS. These data suggest that DORA-22 like the A_2A_R PAMs induced physiologic sleep rather than aberrant sleep in mice with mania-like behavior.

### 2.3 Intraperitoneal administration of diazepam induced aberrant sleep in mice with mania-like behavior

**FIGURE 5:**
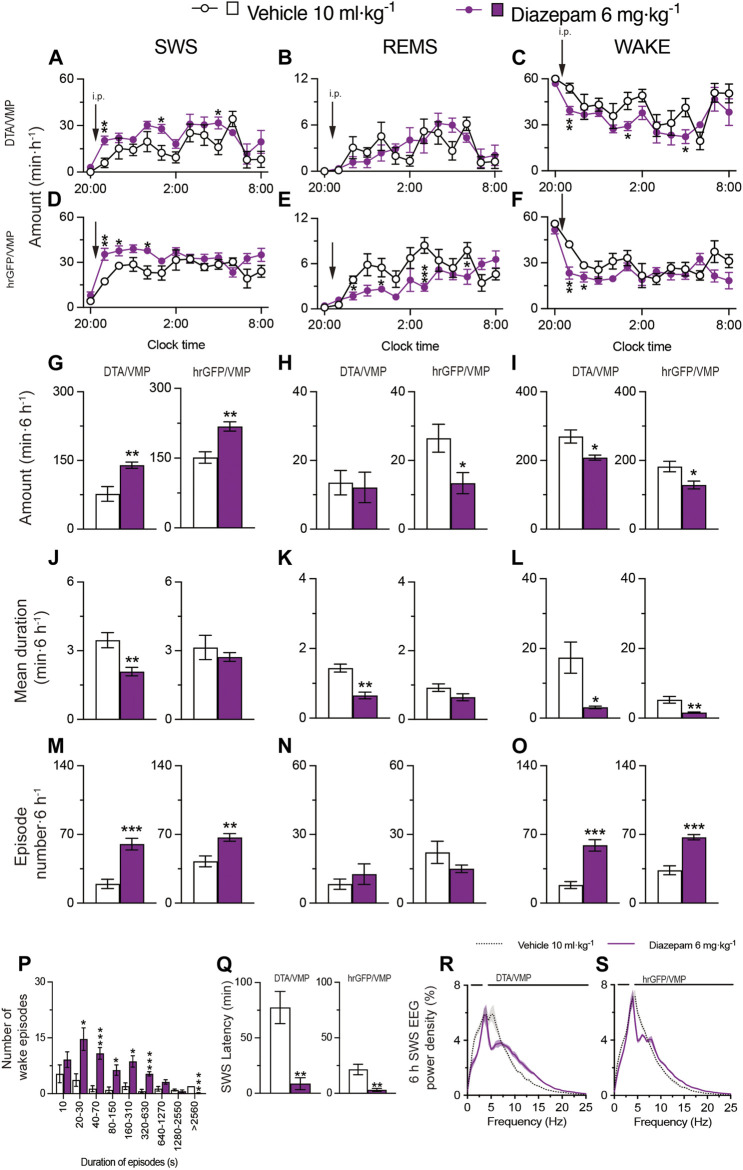
Intraperitoneal injection of benzodiazepine induces sleep in mice with mania-like behavior. **(A–O)** Time course **(A–F)**, total amount **(G–I)**, mean duration **(J–L)**, and episode number **(M–O)** of SWS, REMS, and wakefulness in VGAT-Cre^DTA/VMP^ and VGAT-Cre^hrGFP/VMP^ mice after administering vehicle or diazepam. **(P)** Wake episode spectrum in VGAT-Cre^DTA/VMP^ mice. **(Q)** Latency to sleep onset in VGAT-Cre^DTA/VMP^ and VGAT-Cre^hrGFP/VMP^ mice after administering vehicle or diazepam. **(R,S)** SWS EEG power density for 6 h after injection of vehicle or diazepam into VGAT-Cre^DTA/VMP^ **(R)** and VGAT-Cre^hrGFP/VMP^ **(S)** mice. Bars indicate statistical significance (*p* < 0.05). Data (n = 6/group) are presented as mean ± SEM. Significance levels are indicated as *p < 0.05, **p < 0.01, and ***p < 0.001.

Based on a previously published study (Yu et al., 2021), we next tested the effect of administering 6 mg kg^−1^ diazepam (i.p.) on sleep/wake behavior in mice with mania-like behavior and control mice (Figure 5). We analyzed EEG and EMG recordings made after vehicle or diazepam injections during the dark period at 21:00. SWS was increased for 6 h after the diazepam injections in VGAT-Cre^DTA/VMP^ mice (Figure 5A, SWS: *F*_(1, 10)_ = 9.825, *p* = 0.0106, ANOVA; Figure 5G left, *p* = 0.0022, Mann-Whitney U test) and VGAT-Cre^hrGFP/VMP^ mice (Figure 5D, SWS: *F*_(1, 10)_ = 18.08, *p* = 0.0017, ANOVA; Figure 5G right, *p* = 0.0087, Mann-Whitney U test) compared with vehicle treatment. Interestingly, while diazepam treatment did not affect REMS in mice with mania-like behavior (VGAT-Cre^DTA/VMP^ mice), REMS was decreased for more than 6 h after diazepam injection in the VGAT-Cre^hrGFP/VMP^ mice (Figure 5E, REMS: *F*_(1, 10)_ = 9.285, *p* = 0.0123, ANOVA; Figure 5H right, *p* = 0.0260, Mann-Whitney U test). Administration of diazepam to the VGAT-Cre^DTA/VMP^ mice decreased the mean episode duration of wake, SWS, and REMS (Figure 5J left, *t*_(10)_ = 3.62, *p* = 0.0047, unpaired *t*-test; Figure 5K left*, p* = 0.0022, Mann-Whitney U test; Figure 5L left, *t*_(10)_ = 3.20, *p* = 0.0235, unpaired *t*-test), whereas the SWS and wake episode numbers were increased (Figure 5M left, *t*_(10)_ = 5.32, *p* = 0.0003, unpaired *t*-test; Figure 5O left, *t*_(10)_ = 5.95, *p* = 0.0001, unpaired *t*-test). Moreover, the SWS and wake episode numbers were increased in VGAT-Cre^hrGFP/VMP^ mice after diazepam administration (Figure 5M right, *t*_(10)_ = 3.62, *p* = 0.0047, unpaired *t*-test; Figure 5O right, *t*_(10)_ = 6.37, *p* < 0.0001, unpaired *t*-test). A shift to shorter wake episodes was observed in VGAT-Cre^DTA/VMP^ mice treated with diazepam (Figure 5P). The latency to sleep onset was significantly decreased in the VGAT-Cre^DTA/VMP^ and VGAT-Cre^hrGFP/VMP^ mice treated with diazepam (Figure 5Q left, *p* = 0.0022, Mann-Whitney U test; Figure 5Q right, *p* = 0.0022, Mann-Whitney U test) compared with vehicle treatment. We also analyzed the normalized EEG power spectrum of SWS in VGAT-Cre^hrGFP/VMP^ and VGAT-Cre^hrGFP/VMP^ mice treated with vehicle or diazepam (Figures 5R,S). The EEG activity of mice with mania-like behavior and control animals was significantly decreased in the 1.5- to 7.5-Hz range and increased in the 7.5- to 25-Hz range when treated with diazepam. These data confirm that diazepam produces aberrant sleep rather than physiologic sleep in mice.

### 2.4 Intraperitoneal administration of A_2A_RPAM-1 induced physiologic sleep in MAP6 (STOP) KO mice

**FIGURE 6:**
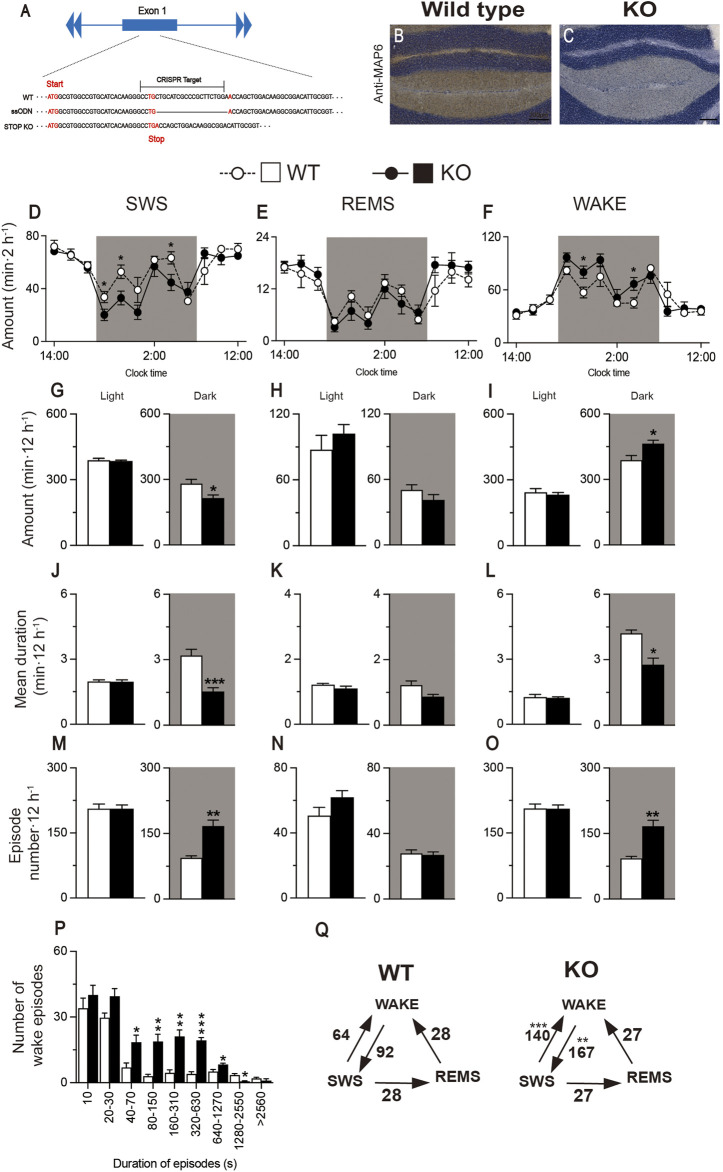
Generation of MAP6 (STOP) knockout (KO) mice with increased wakefulness and fragmented sleep and wake phases. **(A)** Schematic of CRISPR/Cas9-engineered MAP6 gene knockout (KO) using C57BL/6 mice as fertilized egg donors. The CRISPR target including the PAM sequence (total 23 bp) contains part of exon 1 of the MAP6 gene that is located 25 nucleotides after the translation start site. ssODN, single-stranded oligodeoxynucleotide. **(B,C)** Histologic verification of MAP6 KO in the cerebellum of MAP6 (STOP) KO and WT mice. Immunostained MAP6 signals were only detected in wild-type (WT) mice **(B)** and not in MAP6 (STOP) KO mice **(C)**. Scale bar, 200 μm **(D–O)** Baseline time course **(D–F)**, total amount **(G–I)**, mean duration **(J–L)**, and episode number **(M–O)** of SWS, REMS, and wakefulness during the 12h light and 12h dark (shaded areas) phases in MAP6 (STOP) KO and WT mice. **(P)** Wake episode spectrum in MAP6 (STOP) KO and WT mice. **(Q)** Mean number of transitions between SWS, wake, and REMS. Data (n = 6/group) are presented as mean ± SEM. Significance levels are indicated as *p < 0.05, **p < 0.01, and ***p < 0.001.

MAP6 (STOP) null mice, which were previously generated by disrupting exon 1 with a cassette containing lacZ and neo genes, are a genetic mouse model of schizophrenia/psychosis (Andrieux et al., 2002). Impaired glutamatergic transmission in the hippocampus of MAP6 (STOP) null mice is associated with decreased synaptic vesicle density. Moreover, MAP6 (STOP) KO mice have increased dopamine release in the nucleus accumbens (NAc), whereas dopaminergic uptake and auto-inhibition mechanisms are intact (Brun et al., 2005). The association of hippocampal hypo-glutamatergy and limbic hyper-dopaminergy in STOP KO mice provides the intriguing possibility of testing the neuroleptic effects of A_2A_R PAMs in this mouse model. MAP6 (STOP)-null mice sleep less overall and their sleep and wake phases are more fragmented than those of WT mice (Profitt et al., 2016). We generated MAP6 (STOP) KO mice by CRISPR/Cas9 gene editing using WT mice as fertilized egg donors and homologous recombination with a synthetic single-stranded oligodeoxynucleotide (ssODN). The donor ssODN was designed to knock out the MAP6 gene by deleting 20 base pairs (bp) of exon 1 to generate a stop codon 27 nucleotides after the translation start site (Figure 6A). We first histologically confirmed that immunostaining for MAP6 was only detected in WT mice (Figure 6B) and not in MAP6 (STOP) KO mice (Figure 6C). To verify the loss of function *in vivo*, we recorded the baseline sleep/wake behavior of MAP6 (STOP) KO mice for 24 h and compared it with that of WT littermates (Figures 6D–Q). Like the previous MAP6 (STOP)-null mice, CRISPR-engineered MAP6 (STOP) KO mice were more awake in the dark period when mice are more active than during the light period (Figures 6F,I, wake: *F*_(1, 120)_ = 4.323, *p* = 0.0397, ANOVA; Figure 6I right, *t*_(10)_ = 2.89, *p* = 0.0160, unpaired *t*-test). In contrast, SWS, but not REMS, was significantly reduced in the dark period (Figures 6D,E, SWS: *F*_(1, 120)_ = 7.730, *p* = 0.0063, ANOVA; Figure 6G right, *t*_(10)_ = 2.80, *p* = 0.0187, unpaired *t*-test; Figure 6H right, *p* = 0.3095, Mann-Whitney U test). In addition, the sleep and wake phases of MAP6 (STOP) KO mice are more fragmented in the dark than those of WT mice, as evidenced by a decreased duration of SWS or wake episodes (Figure 6J right, *t*_(10)_ = 4.96, *p* = 0.0006, unpaired *t*-test; Figure 6L right, *p* = 0.0152, Mann-Whitney U test), an increased number of SWS and wake episodes, significantly more transitions between SWS and wakefulness (Figure 6M right, *p* = 0.0022, Mann-Whitney U test; Figure 6O right, *p* = 0.0022, Mann-Whitney U test; Figure 6Q, from SWS to wake, *t*_(10)_ = 5.54, *p* = 0.0002, unpaired *t*-test; from wake to SWS, *p* = 0.0022, Mann-Whitney U test), and more wake episodes lasting between 40 and 1270 s (Figure 6P).

**FIGURE 7:**
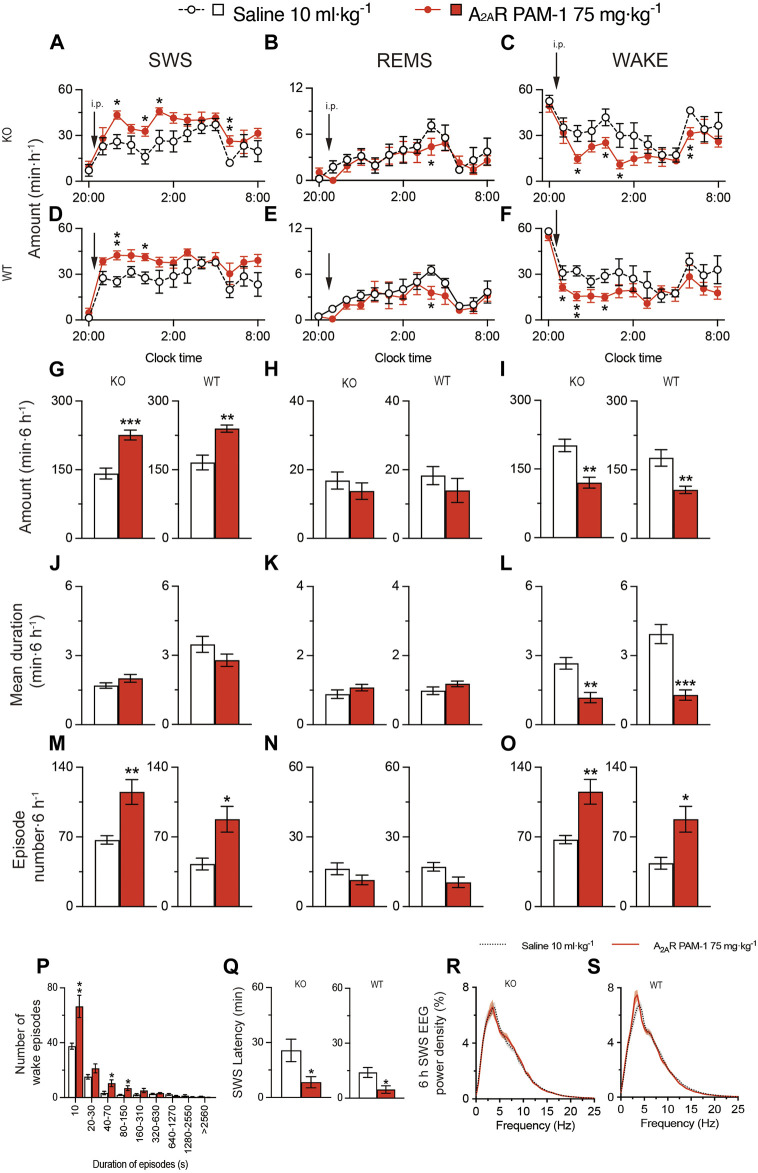
Intraperitoneal injection of A_2A_RPAM-1 induces sleep in a mouse model of schizophrenia. **(A–O)** Time course **(A–F)**, total amount **(G–I)**, mean duration **(J–L)**, and episode number **(M–O)** of SWS, REMS, and wakefulness in MAP6 (STOP) knockout (KO) and wild-type (WT) mice after administering saline or A_2A_RPAM-1. **(P)** Wake episode spectrum in MAP6 (STOP) KO mice. **(Q)** Latency to sleep onset in MAP6 (STOP) KO and WT mice after administering saline or A_2A_RPAM-1. **(R,S)** EEG power density of SWS for 6 h after injecting saline or A_2A_RPAM-1 into MAP6 (STOP) KO **(Q)** and WT **(R)** mice. Data (n = 6/group) are presented as mean ± SEM. Significance levels are indicated as *p < 0.05, **p < 0.01, and ***p < 0.001.

Next, we administered A_2A_RPAM-1 to MAP6 (STOP) KO mice and their WT littermates (Figure 7). For 6 h after injecting A_2A_RPAM-1 (75 mg kg^-1^, i. p.) in MAP6 (STOP) KO mice, SWS was increased, whereas wakefulness was decreased (Figure 7A, SWS: *F*_(1, 10)_ = 23.39, *p* = 0.0007, ANOVA; Figure 7C, wake: *F*_(1, 130)_ = 19.78, *p* < 0.0001, ANOVA). SWS was also increased in WT mice injected with A_2A_RPAM-1 compared with saline, whereas wakefulness was decreased (Figure 7D, SWS: *F*_(1, 10)_ = 14.67, *p* = 0.0033, ANOVA; Figure 7F, wake: *F*_(1, 10)_ = 11.31, *p* = 0.0072, ANOVA). Administration of A_2A_RPAM-1 to MAP6 (STOP) KO and WT mice significantly increased the number of SWS and wake episodes for 6 h after drug injection (Figure 7M left, *t*_(10)_ = 3,64, *p* = 0.0045, unpaired *t*-test; Figure 7M right, *t*_(10)_ = 3.15, *p* = 0.0130 unpaired *t*-test; Figure 7O left, *t*_(10)_ = 3.65, *p* = 0.0044, unpaired *t*-test; Figure 7O right, *t*_(10)_ = 3.09, *p* = 0.0144, unpaired *t*-test), whereas the A_2A_R PAM decreased the mean duration of wake episodes but not the mean duration of SWS episodes (Figure 7J left, *t*_(10)_ = 1.50, *p* = 0.1638, unpaired *t*-test; Figure 7J right, *t*_(10)_ = 1.57, *p* = 0.1466 unpaired *t*-test; Figure 7L left, *t*_*(*10)_ = 4.41, *p* = 0.0013, unpaired *t*-test; Figure 7L right, *t*_(10)_ = 5.59, *p* = 0.0002, unpaired *t*-test). The number of shorter wake episodes lasting between 10 and 150 s increased in MAP6 (STOP) KO mice compared with control mice injected with vehicle (Figure 7P). The REMS behavior of MAP6 (STOP) KO mice and their WT littermates was not affected by A_2A_RPAM-1 administration (Figures 7B,E,H,K,N). The latency to sleep onset was significantly decreased in the MAP6 (STOP) KO and WT mice treated with A_2A_RPAM-1 (Figure 7Q left, *t*_(10)_ = 2.54, *p* = 0.0292, unpaired *t*-test; Figure 7O right, *t*_(10)_ = 2.75, *p* = 0.0206, unpaired *t*-test) compared with vehicle treatment. To determine whether EEG activity was altered by A_2A_RPAM-1 administration, we compared the normalized EEG power spectrum of SWS in MAP6 (STOP) KO and WT mice treated with saline or the A_2A_R PAM (Figures 7R,S). EEG activity in the frequency range of 0.5–25 Hz during SWS was indistinguishable between allosterically modulated and natural (saline injection) SWS.

These data suggest that A_2A_RPAM-1 induces physiologic sleep rather than aberrant sleep in the MAP6 (STOP) KO mice and that positive allosteric A_2A_R modulation can restore SWS in a mouse model of schizophrenia.

### 2.5 Oral administration of the dual orexin receptor antagonist DORA-22 induced physiologic sleep in MAP6 (STOP) KO mice

**FIGURE 8:**
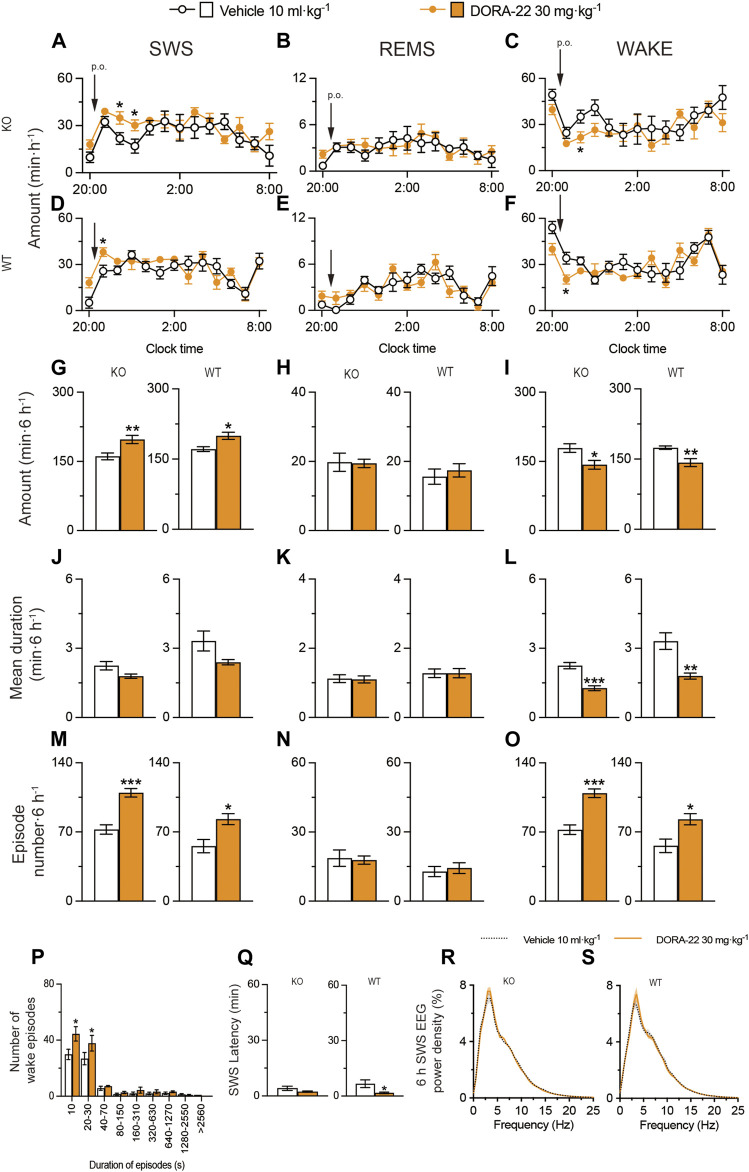
Oral administration of DORA-22 induces sleep in a mouse model of schizophrenia. **(A–O)** Time course **(A–F)**, total amount **(G–I)**, mean duration **(J–L)**, and episode number **(M–O)** of SWS, REMS, and wakefulness in MAP6 (STOP) knockout (KO) and wild-type (WT) mice after administering vehicle or DORA-22. **(P)** Wake episode spectrum in MAP6 (STOP) KO mice. **(Q)** Latency to sleep onset in MAP6 (STOP) KO and WT mice after administering vehicle or DORA-22. **(R,S)** EEG power density of SWS for 6 h after administering vehicle or DORA-22 into MAP6 (STOP) KO **(Q)** and WT **(R)** mice. Data (n = 6/group) are presented as mean ± SEM. Significance levels are indicated as *p < 0.05, **p < 0.01, and ***p < 0.001.

We also administered DORA-22 to MAP6 (STOP) KO mice and their WT littermates (Figure 8). SWS was moderately increased for 6 h after administering 30 mg kg^−1^ DORA-22 (p.o.) in MAP6 (STOP) KO (Figure 8A, SWS: *F*_(1, 130)_ = 8.050, *p* = 0.0053, ANOVA; Figure 8G left, *t*_(10)_ = 3.19, *p* = 0.0097, unpaired *t*-test) and MAP6 WT (Figure 8D SWS: *F*_(12, 130)_ = 1.972, *p* = 0.0318, ANOVA; Figure 8G right, *t*_(10)_ = 3.10 *p* = 0.0112, unpaired *t*-test) mice. In contrast, wakefulness was decreased in MAP6 (STOP) KO (Figure 8C, wake: F (1, 130) = 6.612, *p* = 0.0113, ANOVA; Figure 8I left, t (10) = 2.72, *p* = 0.0215, unpaired *t*-test) and MAP6 WT (Figure 8F, wake: F (12, 130) = 2.207, *p* = 0.0148, ANOVA); Figure 8I right, t (10) = 3.45, *p* = 0.0062, unpaired *t*-test) mice. Administration of DORA-22 to MAP6 (STOP) KO and WT mice significantly increased the number of SWS (Figure 8M left, *t*_(10)_ = 5.71, *p* = 0.0002, unpaired *t*-test; Figure 8M right, *t*_(10)_ = 3.12, *p* = 0.0112, unpaired *t*-test) and wake (Figure 8O left, *t*_*(10)*_ = 5.68, *p* = 0.0002, unpaired *t*-test; Figure 8O right, *p* = 0.0195, Mann-Whitney U test) episodes for 6 h after drug injection, whereas it decreased the mean duration of wake episodes (Figure 8L left, *t*_*(10)*_ = 5.66, *p* = 0.0002, unpaired *t*-test; Figure 8L right, *t*_*(10)*_ = 3.96, *p* = 0.0067, unpaired *t*-test) but not the mean duration of SWS episodes (Figure 8J left, *t*_(10)_ = 2.21, *p* = 0.0518, unpaired *t*-test; Figure 8J right, *t*_(10)_ = 2.04, *p* = 0.0898). The number of shorter wake episodes lasting between 10 and 30 s increased in MAP6 (STOP) KO mice compared with control mice injected with vehicle (Figure 8P). The REMS behavior of MAP6 (STOP) KO mice and their WT littermates was not affected by DORA-22 administration (Figures 8B,E,H,K,N). The latency to sleep onset was significantly decreased only in the WT (Figure 8Q right, *p* = 0.0108 Mann-Whitney U test) and not MAP6 KO (Figure 8Q left, *p* = 0.1342, Mann-Whitney U test) mice treated with DORA-22 compared with vehicle treatment. To determine whether EEG activity was altered by DORA-22 administration, we compared the normalized EEG power spectrum of SWS in MAP6 (STOP) KO and WT mice treated with vehicle or DORA-22 (Figures 8R,S). EEG activity in the frequency range of 0.5–25 Hz during SWS was indistinguishable between DORA-22–induced and natural (vehicle injection) SWS.

These data suggest that DORA-22 induces physiologic sleep rather than aberrant sleep in the MAP6 (STOP) KO mice and that the dual orexin receptor antagonist can restore SWS in a mouse model of schizophrenia, although to a lesser extent than positive allosteric A_2A_R modulation.

## 3 Discussion

Our results suggest that enhancing A_2A_R signaling by intraperitoneal treatment with A_2A_R PAMs suppresses insomnia associated with psychiatric disorders such as mania or schizophrenia in mice. Consequently, allosteric modulation of A_2A_R could be a new therapeutic avenue for insomnia associated with bipolar disorder or psychosis.

Bipolar disorder is a serious mental illness causing unusual and severe mood swings. Individuals with bipolar disorder may experience ‘highs’ (known as mania) and ‘lows’ (known as depression) lasting days to weeks. Increased risk-taking behavior is an important clinical characteristic of bipolar disorder and is associated with maladaptive behaviors such as substance abuse and functional impairment. Insomnia occurs in 70% of bipolar patients even when their mood is stable, and these sleep problems carry an increased risk for further episodes of mania and depression (Harvey et al., 2015). Some clinicians are generous with hypnotics such as benzodiazepines and Z-drugs, while others prefer the lower abuse potential of sedating antidepressants such as trazodone. Ablating GABAergic neurons in the VMP area induces mania-like behaviors in mice, including hyperactivity, antidepressive behaviors, reduced anxiety, increased risk-taking behaviors, distractibility, and an extremely shortened sleep time, by inducing hyperdopaminergic activity in the limbic system (Takata et al., 2018; Honda et al., 2020). Therefore, VMP-ablated mice could be a model of bipolar disorder to study the neuroleptic effects of A_2A_R PAMs and other drugs.

Adenosine is an endogenous somnogen and A_2A_R-expressing NAc indirect pathway neurons regulate sleep (Lazarus et al., 2011; Oishi et al., 2017; Zhou et al., 2019). Therefore, A_2A_R stimulation could be considered a potential therapeutic avenue for insomnia. A_2A_R agonists have potent sleep-inducing effects (Satoh et al., 1999; Scammell et al., 2001; Urade et al., 2003; Methippara et al., 2005), but they also have adverse cardiovascular consequences and therefore, cannot be utilized in clinical sleep medicine. In addition, the development of adenosine analogs as agonists for treating central nervous system disorders such as insomnia, is impeded by poor drug transport across the blood-brain barrier. A_2A_RPAM-1, which is permeable to the blood-brain barrier, can enhance A_2A_R signaling in the brain, thereby inducing sleep (Korkutata et al., 2017; Korkutata et al., 2019). Surprisingly, A_2A_RPAM-1 does not exhibit the typical adverse cardiovascular and body temperature effects of A_2A_R agonists. A_2A_RPAM-1 selectively increases cyclic adenosine monophosphate (cAMP) in A_2A_R-expressing CHO cells only in the presence of adenosine and induces extended SWS, but not REMS, in WT mice but not in A_2A_R KO animals. A_2A_RPAM-1 does not affect body temperature, cardiac function, or blood pressure, unlike A_2A_R agonists (Korkutata et al., 2019), suggesting that adenosine or A_2A_R expression levels in the cardiovascular system are not sufficient to trigger an A_2A_RPAM-1 response under normal physiologic conditions. In this study, we found that A_2A_RPAM-1 and its derivative A_2A_RPAM-2, which has a higher A_2A_R PAM efficiency than A_2A_RPAM-1, strongly induced SWS in mice with mania-like behavior, similar to DORA-22 and more efficiently than diazepam. A_2A_R PAMs likely induce SWS by enhancing activation of A_2A_Rs on NAc indirect pathway neurons that inhibit wake-promoting GABAergic neurons in the ventral pallidum (VP) (Oishi et al., 2017). VP GABAergic neurons promote arousal by innervating the ventral tegmental area (VTA), resulting in disinhibition of VTA dopaminergic neurons, and innervating the lateral hypothalamus, resulting in disinhibition of orexin neurons (Li et al., 2021). The mesolimbic VTA-NAc-VP loop, which acts as an “amplifier” of dopaminergic signaling, together with orexin neurons, which can induce cortical arousal, constitutes a powerful arousal system. It is therefore not surprising that the A_2A_R PAMs, which have no antagonistic activity at orexin receptors (Figures 1D,E), and dual orexin receptor antagonists have similar effects in mice. In addition, analysis of the EEG power spectrum showed that the A_2A_R PAMs induced psychologic SWS rather than aberrant SWS, as was observed with diazepam. Interestingly, A_2A_R PAMs and DORA-22 also induced REMS in mice with mania-like behaviors, whereas only DORA-22 increased REMS in the control mice (WT mice). Thus, an A_2A_R PAM could improve aberrant SWS and REMS in patients with bipolar disorder.

Insomnia is also a hallmark of the prodromal phase of psychoses such as schizophrenia and a major risk factor for impending relapse of psychosis. Treatment of insomnia might play a role in the prevention and treatment of psychosis (Keshavan et al., 2004; Manber et al., 2008). Only a few clinical trials have specifically examined the treatment of insomnia in schizophrenia patients because patients with psychiatric disorders such as schizophrenia are typically not included in clinical trials for insomnia therapies (Roehrs et al., 2018). Therefore, the best way to treat insomnia in schizophrenia is unknown, but paliperidone, melatonin, and eszopiclone are often used to treat insomnia in people with schizophrenia. Mice deficient in MAP6 (STOP) have cognitive, behavioral, and neurobiologic deficits similar to those of schizophrenia patients (Volle et al., 2013). MAP6 (STOP)-null mice sleep less, especially during the dark phase when they are mainly active, and their sleep and wake phases show more fragmentation than those of WT mice (Profitt et al., 2016). We generated a CRISPR/ssODN-engineered KO of MAP6 (STOP) with aberrant sleep/wake behavior like that of the original MAP6 (STOP)-null mice. The A_2A_RPAM-1 had a potent hypnotic effect in MAP6 (STOP) KO mice and efficiently prevented the disruption of sleep/wake patterns in the KO mice.

In addition, positive allosteric modulators of A_2A_Rs may also alleviate other symptoms of schizophrenia. Psychotic symptoms such as delusions are caused by impaired discrimination of environmental stimuli. Recent evidence showed that dopamine D_2_ receptors (D_2_Rs) mediate discrimination learning in the NAc, but A_2A_Rs expressed together with D_2_Rs in the NAc are required for discrimination learning. While normal mice can discriminate between reward-predictive and non-reward–predictive tones several days after generalized reward conditioning (when any tone is reward-predictive), mice in which A_2A_Rs are blocked in the NAc do not show this ability (Iino et al., 2020). In addition, hypofunction of N-methyl-D-aspartate-type glutamate receptors is thought to be involved in schizophrenia, as N-methyl-D-aspartate receptor antagonists such as phencyclidine and dizocilpine, also known as MK-801, cause psychotic and cognitive disorders in humans and animals (Field et al., 2011). Deleting A_2A_Rs in NAc astrocytes leads to motor and memory impairments relevant to schizophrenia, namely, exacerbation of the MK-801-induced psychomotor response and impaired working memory (Matos et al., 2015). Thus, enhancing A_2A_R signaling may contribute to the treatment of sleep disorders and psychosis, the latter of which will be studied in MAP6 (STOP) KO mice in the future.

In conclusion, the sleep-promoting effects of A_2A_R PAMs open the doors for the potential therapeutic use of these chemicals for treating diseases. Allosteric modulators exert their effects only where and when the orthosteric ligand is released, conferring a therapeutic advantage over classical agonist and antagonist drugs. Thus, allosteric A_2A_R modulation could provide patients with an effective and safe treatment of various diseases. Adenosine, for example, is present in high concentrations in areas of inflammation due to cell activation and breakdown (Martin et al., 2000; Sperlágh et al., 2000; Sottofattori et al., 2001), and A_2A_Rs are responsible for the anti-inflammatory effects of adenosine (Sitkovsky, 2003; Haskó and Cronstein, 2004). Thus, A_2A_R PAMs may also represent a potential therapeutic approach to inflammation.

## 4 Materials and methods

### 4.1 Animals

VGAT-Cre with a C57BL/6 and 129 mixed background (Vong et al., 2011), kindly provided by Dr. Bradford Lowel (Harvard Medical School, Boston, MA) and MAP6 KO (newly generated in this study) mouse strains were used in this study. Male mice (12–20 weeks of age, 20–30 g) used in the experiments were single-housed in insulated sound-proof chambers maintained at an ambient temperature of 23°C ± 0.5°C with 55% ± 3% humidity on a 12-h light/dark cycle (lights on at 8:00). Food and water were provided *ad libitum*. All experiments were performed in accordance with the Animal Care Committee of the University of Tsukuba, and every effort was made to minimize the number of animals used as well as any pain or discomfort.

### 4.2 A_2A_RPAM-2 synthesis

A 5-mL microwave vial was charged with 4-bromo-2-pyridinecarboxylic acid (0.10 g, 0.498 mmol), 4-propylaniline (70.8 μL, 0.498 mmol), lithium amide (57.1 mg, 2.49 mmol), and 1,4-dioxane (5 mL). The vial was heated in the microwave for 5 min at 200°C and thereafter, cooled to room temperature. The reaction mixture was quenched by adding a solution of saturated aqueous ammonium chloride (30 mL) and extracted with chloroform (4 × 20 mL). The combined organic layers were washed with brine, dried over anhydrous sodium sulfate, and concentrated *in vacuo*. The residue was purified by silica gel column chromatography (chloroform/10% acetic acid in methanol [100/0 → 90/10]) to give 4-((4-propylphenyl)amino)picolinic acid (A_2A_RPAM-2, 76.3 g, 60%, Figure 1A) as a brown solid: IR (KBr) 3034, 2956, 1666, 1546, 1391, 1295, 791 cm^−1^; ^1^H NMR (400 MHz, CD_3_OD) δ 0.97 (t, 3H, *J* = 7.3 Hz), 1.68 (tq, 2H, *J* = 7.3, 7.3 Hz), 2.66 (t, 2H, *J* = 7.3 Hz), 7.26 (dd, 1H, *J* = 2.3, 7.3 Hz), 7.32 (d, 2H, *J* = 8.2 Hz), 7.37 (d, 2H, *J* = 8.2 Hz), 7.48 (d, 1H, *J* = 2.3 Hz), 8.32 (d, 1H, *J* = 7.3 Hz); ^13^C NMR (100 MHz, CD_3_OD) 14.1, 25.7, 38.5, 124.5, 127.6, 128.8, 129.7, 130.9, 131.5, 137.3, 141.5, 147.5, 166.6; Anal. Calcd for C_15_H_16_N_2_O_2_: C, 69.80; H, 6.33; N, 10.85; O, 13.02. found: C, 69.72; H, 6.40; N, 10.91; O, 12.97.

The sodium salt of A_2A_RPAM-2 for intraperitoneal injections was prepared as previously described for A_2A_RPAM-1 (Korkutata et al., 2019).

### 4.3 cAMP assay

Activation of A_2A_Rs was quantified on the basis of cAMP accumulation in CHO cells expressing mouse A_2A_Rs. CHO cells were suspended in Hank’s balanced salt solution (HBSS) containing 1M HEPES and 0.25M 3-isobutyl-1-methylxanthine in 384-well micro-plates (2×10^3^ cells/well) and incubated with adenosine and A_2A_R PAM-1 or A_2A_RPAM-2 at the indicated concentrations for 30 min at 25°C. After adding the detection mixture containing the Eu-cAMP tracer and ULight-anti-cAMP antibody, the plates were further incubated for 1 h at 25°C. A microplate reader (ARVO X5, PerkinElmer, Waltham, MA; excitation: 340 nm; emission: 665 nm) was used to measure the Förster resonance energy transfer (FRET) signal. All experiments were performed according to the manufacturer’s instructions (LANCE Ultra cAMP Kit, PerkinElmer). The cAMP levels are based on the dynamic range (“linear portion”) of the cAMP standard curve and normalized to the baseline or adenosine-treated group.

### 4.4 Calcium assay

CHO cells stably expressing human OX_1_Rs or OX_2_Rs were seeded in a 96-well-plate (1×10^4^ cells/well) and incubated with 5% fetal bovine serum/Dulbecco’s modified Eagle medium at 37 °C for 48 h. The cells were then loaded with 4 μM fluorescent calcium indicator Fura 2-AM (Cayman Chemical) in HBSS containing 20 mM HEPES, 2.5 mM probenecid, 5% CremophorEL, and 0.1% bovine serum albumin and incubated for 1 h at 37°C. Next, the cells were washed once, and 50 μL of HBSS buffer was added. Cells were pretreated with 25 μL of different concentrations of A_2A_RPAM-1 or A_2A_RPAM-2 for 15 min and 25 μL 0.3 nM OXA (Peptide Institute) was added to the cells. The increase in the intracellular calcium concentration was measured from the ratio of emission fluorescence at 510 nm to excitation fluorescence at 340 or 380 nm using the Functional Drug Screening System 7000 (Hamamatsu Photonics).

### 4.5 UPLC-MS/MS analysis

The brains of mice injected intraperitoneally with A_2A_RPAM-1 at 21:00 were collected 1 h after treatment. Each brain was combined with 300 μL of acetonitrile with 1% formic acid (v/v) and vortexed for 3 min. Then, the precipitated proteins were removed by centrifugation (3000 rpm for 5 min), and the supernatant was transferred to a HybridSPE-Phospholipid Ultra cartridge (Supelco) and eluted from the cartridge by applying a vacuum. The eluate was injected into a Waters ACQUITY UPLC-MS/MS system with an electrospray ionization interface operating in negative ion mode. An ACQUITY UPLC BEH C18 column (1.7 μm, 50 mm × 2.1 mm; Waters) with a graded acetonitrile/water mobile phase at a flow rate of 500 μL/min was used for UPLC separation. A_2A_RPAM-1 was detected by single-ion (m/z 392) monitoring.

### 4.6 Stereotaxic AAV injection and placement of EEG/EMG electrodes

The mice used in the behavioral experiments were anesthetized with isoflurane (4% for induction, 2% for maintenance) for brain microinjection and EEG/EMG electrode implantation. To selectively ablate GABAergic neurons in the VMP, VGAT-Cre mice were injected bilaterally into the VMP (3.4 mm posterior and 0.2 mm lateral to bregma, 4.4 mm below the dura) with AAV-FLEX-DTA (120 nL, 8.6 × 10^10^ particles ml^-1^) or AAV-FLEX-hrGFP (120 nL, 1.5 × 10^11^ particles ml^-1^) using a glass micropipette and an air pressure injector system (Takata et al., 2018). For monitoring sleep/wake behavior, the mice were chronically implanted with EEG and EMG electrodes for polysomnography (Oishi et al., 2016). As EEG electrodes, 2 stainless steel screws were implanted into the skull. As EMG electrodes, 2 insulated Teflon-coated silver wires were placed bilaterally into the trapezius muscles. The electrodes were fixed to the skull using dental acrylic.

### 4.7 EEG/EMG polygraphic recordings and pharmacologic treatment

EEG/EMG recordings were performed as previously described (Oishi et al., 2016). Briefly, after allowing 1–2 weeks for postoperative recovery and transgene expression, the mice were connected to EEG/EMG recording cables. The EEG/EMG signals were amplified and filtered by an amplifier (Biotex, Kyoto, Japan; EEG: 0.5–64 Hz, EMG: 16–64 Hz), digitized at a sampling rate of 128 Hz, and recorded using SLEEPSIGN software (Kissei Comtec, Matsumoto, Japan). Vigilance states were scored offline by characterizing 10-s epochs into three stages: awake, SWS, and REMS according to standard criteria (Oishi et al., 2016).

A_2A_RPAM-1, synthesized as previously described (Korkutata et al., 2019), A_2A_RPAM-2, and diazepam (Merck, Darmstadt, Germany) were intraperitoneally injected. The A_2A_RPAM-1 and A_2A_RPAM-2 were dissolved in saline, whereas diazepam was dissolved in saline with 10% dimethyl sulfoxide and 2% (w/v) cremophor. DORA-22 [(2R, 5R)-5-[(5-fluoropyridin-2-yl)oxymethyl]-2-methylpiperidin-1-yl]-(5-fluoro-2-pyrimidin-2-ylphenyl)methanone was kindly provided by Merck and Co. and administered orally. Vit-E TPGS (D-α-tocopherol polyethylene glycol 1000 succinate) was used as a vehicle for DORA-22. A 20% Vit-E TPGS was dissolved in deionized water by placing over a magnetic stirrer overnight with mild heat (40°C–45°C). Specific doses used in each experiment are reported in the Results section and figures. For pharmacologic experiments, mice were treated with the vehicle to obtain control data. After a 24-h baseline recording, mice were administered vehicle or drugs intraperitoneally or orally as indicated at 21:00 on different days.

### 4.8 Histology

For histologic analyses, the mice were deeply anesthetized with an overdose of chloral hydrate (500 mg kg^-1^, i. p.) and perfused through the left ventricle of the heart with saline followed by neutral buffered 10% formalin. The brains were removed and placed in 20% sucrose in phosphate-buffered saline overnight at 4°C to reduce freezing artifacts. The brains were then sectioned at 40 μm on a freezing microtome. For *in situ* hybridization, a 918-bp digoxigenin-labeled riboprobe for VGAT mRNA was generated by PCR amplification with the forward (5′-GCA​TGT​TCG​TGC​TGG​GCC​TAC​C-3′) and reverse (5′-CAG​CGC​AGC​GTC​AGC​CCC​CAG-3′) primers conjugated with the T7 promoter using mouse tail genomic DNA (gDNA) as a template, followed by *in vitro* transcription. The brain sections were then incubated with 1 μg mL^-1^ of the VGAT probe in 5X sodium citrate buffer containing 50% formamide at 50°C overnight, washed in 1X saline sodium citrate buffer at 50°C, incubated with alkaline phosphatase-conjugated anti-digoxigenin antibody (1:500, Roche, Mannheim, Germany) overnight, and visualized by reaction with 5-bromo-4-chloro-3-indolyl phosphate/nitro blue tetrazolium (BCIP/NBT, Merck, Darmstadt, Germany).

Immunohistochemistry for MAP6 (STOP) was performed on free-floating sections that were incubated in 0.3% hydrogen peroxide for 30 min. Following antigen retrieval using sodium citrate Heat-Induced-Epitope-Retrieval buffer (BioLegend, San Diego, CA) for 15 min at 99°C, the tissue was incubated overnight at 4°C with a mouse anti-MAP6 (STOP) antibody (1:100; BioLegend). Sections were then treated with avidin-biotin complex (1:1000; Vectastain ABC Elite kit; Vector Laboratories, Newark, CA) for 1 h, and immunoreactive cells were visualized by reaction with 3,3′-diaminobenzidine and 0.1% hydrogen peroxide.

### 4.9 Generation of MAP6 (STOP) KO mice by CRISPR/Cas9 gene editing

To generate the CRISPR-engineered MAP6 (STOP) KO mice, guide RNA (gRNA), ssODN, Cas9 protein (Thermo Fisher Scientific, Waltham, MA), and fertilized eggs of female C57BL6/J mice (Charles River, Cambridge, MA) were used. The gRNA (5′-CCT​GCT​GCA​TCG​CCC​GCT​TCT​GG-3′) was synthesized using a GeneArt Precision gRNA Synthesis Kit (Thermo Fisher Scientific). The donor ssODN was designed to knock out the MAP6 gene (NM 010837: variant 1, also known as N-STOP) by deleting 20 bp of exon 1 to generate a stop codon 27 nucleotides after the translation start site. The 100-nt ssODN consisting of nucleotides 555 to 674 of exon 1 without the deleted nucleotides 605-624 (5′-CTG​CAT​CGC​CCG​CTT​CTG​GA-3′) was synthesized by Fasmac (Atsugi, Japan). Female C57BL6/J mice were injected with 5 IU pregnant mare serum gonadotropin and 5 IU human chorionic gonadotropin at a 48-h interval and mated. Fertilized 1-cell embryos were collected from the oviducts, and gRNA, ssODN, and Cas9 protein were microinjected into the pronuclei of these embryos. The embryos were then transferred into pseudopregnant ICR mice for F0 mouse production. Direct sequencing from the tail genomic DNA of F0 mice was performed to confirm the deletion of the nucleotides. For later genotyping, the tail genomic DNA was amplified with the forward (5′-ACT​TTA​CGG​ACT​TTA​TCT​CAG​CG-3′) and the reverse (5′-AAC​TGC​ATC​CGA​CTC​TCC​C-3′) primers to identify MAP6 KO mice and WT littermates (KO: 381 bp, WT: 401 bp). The latter primer can be used in conjunction with a different forward primer (5′-CTG​CAT​CGC​CCG​CTT​CTG-3′) to verify the genomic DNA deletion in exon 1 (KO: no amplicon, WT: 196 bp).

### 4.10 Statistical analysis

Statistical analysis was performed using Graph Pad Prism 7.0 (GraphPad Software). All data were subjected to the Kolmogorov-Smirnov test for Gaussian distribution and variance. Comparisons between 2 groups were performed using the unpaired 2-tailed Student’s t-test or 2-tailed Mann-Whitney U test. Comparisons among multiple parameters were performed by a 2-way repeated-measures Analysis of Variance (ANOVA) followed by Sidak’s *post hoc* comparisons. Significance levels in the figures are represented as *p < 0.05, **p < 0.01, and ***p < 0.001. Error bars in the graphs represent mean ± SEM.

## Data Availability

The raw data supporting the conclusion of this article will be made available by the authors, without undue reservation.
